# Mitochondrial Calcium Signaling in Pancreatic β-Cell

**DOI:** 10.3390/ijms22052515

**Published:** 2021-03-03

**Authors:** Anna Weiser, Jerome N. Feige, Umberto De Marchi

**Affiliations:** 1 Nestlé Research—EPFL Innovation Park, CH-1015 Lausanne, Switzerland; anna.weiser@rd.nestle.com (A.W.); jerome.feige@rd.nestle.com (J.N.F.); 2 Molecular Nutritional Medicine, Else Kröner Fresenius Center for Nutritional Medicine, Technische Universität München, 85354 Freising, Germany

**Keywords:** mitochondria, calcium, insulin secretion, β-cell, diabetes

## Abstract

Accumulation of calcium in energized mitochondria of pancreatic β-cells is emerging as a crucial process for pancreatic β-cell function. β-cell mitochondria sense and shape calcium signals, linking the metabolism of glucose and other secretagogues to the generation of signals that promote insulin secretion during nutrient stimulation. Here, we describe the role of mitochondrial calcium signaling in pancreatic β-cell function. We report the latest pharmacological and genetic findings, including the first mitochondrial calcium-targeted intervention strategies developed to modulate pancreatic β-cell function and their potential relevance in the context of diabetes.

## 1. Introduction

Pancreatic β-cells constitute a small endocrine tissue organized, together with other endocrine cells, in the islets of Langerhans, scattered throughout the exocrine tissue of the pancreas [1]. β-cells sense glucose and secrete insulin in order to lower blood glucose levels after a meal. Defective insulin secretion underlies diabetes mellitus, which is a metabolic disorder characterized by elevated blood glucose levels [2,3]. The WHO’s first global report on diabetes indicates that the number of adults living with diabetes has almost quadrupled since 1980 to 422 million adults. This dramatic increase is largely due to the rise in Type 2 diabetes, whose driving factors include overweight and obesity. This disease develops when the β-cells of the endocrine pancreas fail to secrete sufficient hormones to compensate for the insulin resistance in the peripheral target tissues, liver, muscle and fat [4]. Diabetes is a non-communicable disease for which new approaches to prevention and treatment urgently need to be found. Targeting pancreatic β-cells is a promising strategy for the treatment of diabetes, due to the crucial role of the pancreatic β-cell in the pathogenesis of both Type 1 and Type 2 diabetes [5]. Therefore, preservation, expansion or improved function of β-cells are current approaches for targeting this cell type in the management of diabetes. Modulation of the biological pathways that regulate β-cell function represents the next stage of discovery in this field [5].

In this context, targeting mitochondrial Ca^2+^ represents an innovative approach to modulate β-cell function and to potentially promote beneficial effects for diabetic patients. Thus, dysregulation of Ca^2+^ signaling has been reported to have profound effects on β-cell performance and to increase the risk of developing diabetes [6,7]. Furthermore, modulation of dynamic cellular Ca^2+^ homeostasis has been proposed to prevent cytokine-mediated β-cell loss in diabetes [8]. In the pancreatic β-cell, Ca^2+^ homeostasis and β-cell function are substantially linked to mitochondrial function [9]. Therefore, mitochondria play a key role in β-cells during nutrient stimulation by linking the metabolism of glucose and other secretagogues to the generation of signals that promote insulin secretion [9]. Diabetes causes marked inhibition of mitochondrial metabolism in pancreatic β-cells [10]. Mitochondria are versatile intracellular organelles that are able to take up and release calcium [11,12]. Mitochondrial matrix Ca^2+^ is an activating signal for insulin secretion, and its requirements for signal-dependent hormone secretion have been highlighted [13]. Recently, the molecular identity of the mitochondrial Ca^2+^ uniporter (MCU), the transporter that mediates mitochondrial calcium uptake, has been revealed [14]. Genetic and pharmacological evidence has demonstrated the crucial role of mitochondrial Ca^2+^ in modulating pancreatic β-cell signal transduction, opening new perspectives for intervention [15,16,17].

Excellent reviews on the bioenergetic role of mitochondria and mitochondrial Ca^2+^ in metabolism–secretion coupling in the pancreatic β-cell are available [9,18,19], including the control of mitochondrial structure and function by calcium [20]. The role of mitochondrial ion channels in the pathophysiology of the pancreatic β-cell has also been described recently [21]. In this paper, we focus on the role of mitochondrial Ca^2+^ in pancreatic β-cell signal transduction. We report the latest pharmacological and genetic evidence, including the first intervention strategy targeting mitochondrial Ca^2+^ in the β-cell.

## 2. Pancreatic β-Cell Signal Transduction and Ca^2+^ Homeostasis

**Figure 1:**
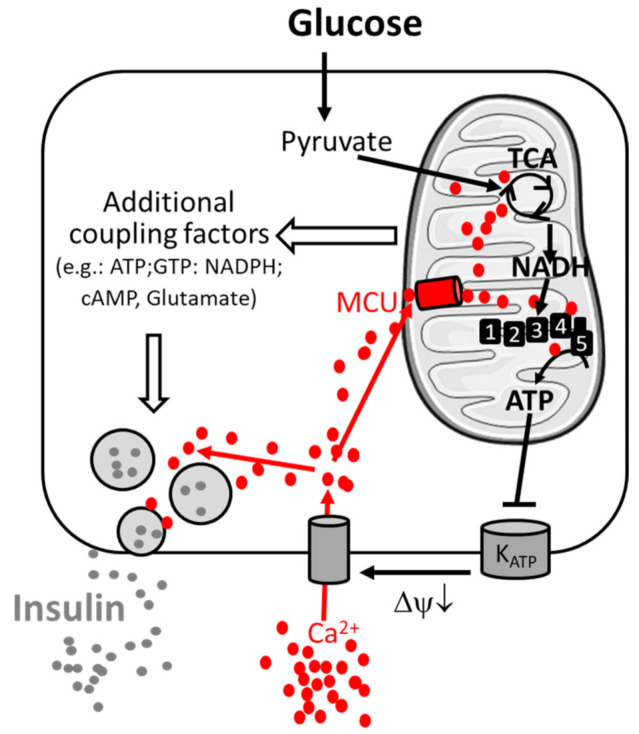
Consensus model of the signal transduction pathway of pancreatic β-cells. Metabolism–secretion coupling of β-cells requires the sequential activation of glycolysis, mitochondrial oxidative metabolism and Ca^2+^ entry through the plasma membrane. Glucose stimulates glycolysis and pyruvate production. Pyruvate triggers mitochondrial metabolism and the formation of the reduced form of nicotinamide adenine dinucleotide NADH (by the TCA cycle), which is the fuel for the respiratory complexes (1,2,3,4), enabling ATP production by ATP-synthase (5). ATP inhibits the K_ATP_ channel, inducing membrane depolarization (ΔΨ↓) and Ca^2+^ entry through voltage-gated Ca^2+^ channels, promoting insulin secretion. Ca^2+^ is taken up in parallel by mitochondria via the mitochondrial Ca^2+^ uniporter (MCU) and facilitates sustained insulin secretion. The amplifying pathway of metabolism–secretion coupling is co-generated by additive coupling factors. Additional players contributing to Ca^2+^ homeostasis are mentioned in the main text.

In the pancreatic β-cell, metabolism–secretion coupling describes the molecular mechanism linking nutrient sensing and signaling to insulin secretion. This process relates to the consensus model and additional coupling factors (including both triggering and amplifying pathways) of glucose-stimulated insulin secretion [22,23]. Glucose-stimulated insulin secretion is relatively well characterized and requires the sequential activation of several biological processes (Figure 1).

Glucose enters the β-cell by glucose-mediated transporters (GLUT). In the cytosol, it is metabolized by glycolysis to generate pyruvate, which is taken up by mitochondria. Mitochondrial pyruvate is metabolized by the tricarboxylic acid (TCA) cycle, which generates reducing equivalents NADH (reduced nicotinamide adenine dinucleotide) and FADH2 (reduced flavin adenine dinucleotide), which are substrates of the mitochondrial respiratory chain. Activation of mitochondrial respiration leads to mitochondrial ATP synthesis and thus to an increased cytosolic ATP-to-ADP ratio, which induces the closure of plasma membrane K_ATP_ channels and promotes plasma membrane depolarization. This opens voltage-gated plasma membrane Ca^2+^ channels, leading to an increase in cytosolic Ca^2+^ concentration, which finally triggers insulin exocytosis by activating Ca^2+^-sensitive granule-resident proteins (e.g., synaptotagmin-7 [24]). The amplifying pathways of metabolism–secretion coupling are contributed by additive coupling factors, and mitochondria have been characterized as a source of coupling factors [9,22].

The coupling between nutrient stimulation and hormone secretion is closely linked to Ca^2+^ homeostasis in the pancreatic β-cell [7,25,26,27]. Therefore, insulin secretion is driven by electrical activity and oscillations of intracellular Ca^2+^ concentrations. However, in addition to K_ATP_ channels and voltage-dependent Ca^2+^ channels [28], other plasma membrane channels and intracellular stores have been shown to be involved in insulin secretion in pancreatic β-cells [26,29]. In particular, store-operated Ca^2+^ channels, which are voltage-independent Ca^2+^ channels activated upon depletion of the endoplasmic reticulum Ca^2+^ stores, and transient receptor potential channel 1 (TRPC1), have been indicated to be involved in insulin secretion [30,31]. For store-operated Ca^2+^ channels, Orai1 has been identified as the main protein that conducts the previously described Ca^2+^ release-activated current (I_CRAC_). The activity of Orai1 channels is tightly controlled by the endoplasmic reticulum membrane protein stromal interacting molecule 1 (STIM1), which acts as an endoplasmic reticulum Ca^2+^ sensor and translocates, upon endoplasmic reticulum depletion, to endoplasmic reticulum/plasma membrane regions, where Orai1 is clustered [31]. In addition, mobilization of intracellular Ca^2+^ from the endoplasmic reticulum has been suggested to potentiate glucose-stimulated hormone secretion [32], and the Type 2 ryanodine receptor (RyR2) has been proposed to play a crucial role in regulating insulin secretion and glucose homeostasis [33]. Moreover, an atypical Ca^2+^ leak has been observed in the endoplasmic reticulum, specifically in pancreatic islets and β-cells. This continuous Ca^2+^ efflux from the endoplasmic reticulum was modulated by GSK3β-dependent phosphorylation of presenilin-1 and promoted mitochondrial activation [34,35]. Additional intracellular acidic compartments may contribute to the local modulation of β-cell Ca^2+^ homeostasis and thus β-cell function, including insulin granules and other acidic stores (e.g., lysosomes) [26].

The last player in pancreatic β-cell Ca^2+^ homeostasis and signal transduction is represented by the mitochondrial network. Therefore, mitochondria are intracellular organelles that take up and release Ca^2+^, promoting the sensing and shaping of cytosolic Ca^2+^ signals [36]. The role of mitochondrial Ca^2+^ signaling in energized mitochondria of the pancreatic β-cell is emerging as a biological process of critical importance to pancreatic β-cell function and is highlighted in the next section.

## 3. The Mitochondrial Calcium Uniporter and Its Existence in Pancreatic β-Cells

Despite the undisputed role of cytosolic Ca^2+^ elevation in triggering insulin secretion in the pancreatic β-cell, it is also accepted that such a rise in itself does not sustain insulin secretion [18,37,38]. Therefore, mitochondria have been demonstrated to contribute to robust insulin secretion by triggering additional regulatory factors, and it has been proposed that mitochondrial Ca^2+^ plays a crucial role as a receiver and generator of the signals essential for metabolism–secretion coupling [9,39]. The discovery and molecular definition of the MCU [14,40], the transporter which mediates the transport of Ca^2+^ in the mitochondrial matrix under physiological conditions, is shedding light on the role of mitochondrial Ca^2+^ elevation in different tissues, including the pancreatic β-cell (see Section 4).

The MCU complex is a low-affinity, high-capacity Ca^2+^ uniporter embedded in the inner mitochondrial membrane with an approximate in vitro Ca^2+^ binding affinity estimated to be between 10 and 70 µM [41,42,43,44] in different tissues. The transport of Ca^2+^ ions into the mitochondrial matrix is electrochemically driven by a strong electrical gradient (~180 mV) through the ion-impermeable inner mitochondrial membrane [45]. As reported for many other tissues, in the pancreatic β-cell, resting-state mitochondrial Ca^2+^ levels are also very close to cytosolic Ca^2+^ levels, indicating tight regulation of the MCU [18]. Permeabilized INS-1 cells (a cell-line model of pancreatic insulin-secreting cells) display significant mitochondrial Ca^2+^ uptake when perfused with buffers containing a Ca^2+^ concentration of only 150 nM [46], which is below the general threshold of MCU activation [47]. This study suggests a slightly higher affinity configuration of the β-cell MCU, compared with the MCU of other tissues. However, in permeabilized β-cells perfused with a range of different Ca^2+^ buffers, mitochondrial Ca^2+^ uptake was more efficient above 2 µM [48]. These data indicate that although some studies demonstrated a lower Ca^2+^ activation threshold, the MCU of β-cells also behaves as a low affinity, high capacity Ca^2+^ transport system, with similar properties to those reported in other tissues. In any case, the mitochondrial Ca^2+^ concentration in glucose-stimulated β-cells was reported to reach only 600–800 nM [13,18,49], mirroring cytosolic Ca^2+^ events. Purinergic activation and potassium-induced depolarization generate cytosolic Ca^2+^ transients around one micromolar, with the corresponding mitochondrial Ca^2+^ rises reaching nearly 5 µM [39]. These results indicate that, on average, β-cell mitochondria barely reach micromolar Ca^2+^ concentrations in the matrix during physiological activation.

The MCU complex consists of at least six subunits, each of which plays an individual role in orchestrating mitochondrial Ca^2+^ uptake [41]. The MCU subunit is a 40 kDa protein that, together with its paralog, MCUb [50], has been proposed to form a tetramerizing pore which penetrates the inner mitochondrial membrane [51]. In order to channel Ca^2+^ ions into the mitochondrial matrix, it requires a third subunit known as the essential MCU regulator (EMRE), a 10 kDa protein [50,52]. EMRE is a single-transmembrane protein whose transmembrane helix connects it to the MCU subunit [53]. The MCU and MCUb share 50% sequence similarity and have opposite effects on mitochondrial Ca^2+^ uptake [50]. While the MCU has a promoting effect on mitochondrial Ca^2+^ uptake, MCUb exerts a negative effect on mitochondrial Ca^2+^ uptake [54]. The ratio of MCU and MCUb varies among tissues and is potentially influenced by metabolic impairments, leading to the hypothesis that an altered MCU/MCUb ratio may affect β-cell function [55,56,57]. When Ca^2+^ in the intermembrane space exceeds ~0.6 µM, it initiates activation of the MCU complex via the MCU gatekeeper protein paralogs mitochondrial calcium uptake 1 and 2 (MICU1 and MICU2) [44,58]. MICU2 is a 50 kDa protein and shares approximately 25% sequence identity with the 54 kDa protein MICU1 [42]. Neither MICU1 nor MICU2 contain transmembrane domains that would link them to the MCU subunit, so it has been proposed that MICU1 is linked to the MCU via EMRE through electrostatic interaction [58]. However, a more recent study revealed an additional direct binding of MICU1 to the highly conserved DIME motif of the MCU [59,60]. In addition, another study showed an interaction between MICU1/MICU2 and cardiolipin [58]. Through cysteine residues, MICU2 forms an intermembrane space-facing heterodimer with MICU1 via disulfide bonds [51]. Both gatekeeper proteins contain EF-hand motifs (consisting of a helix E, a loop and another helix F), with a Ca^2+^ binding affinity of ~0.3 µM for MICU1 and a Ca^2+^ binding affinity of ~0.6 µM for the MICU1–MICU2 dimer [58].

Genetic ablation of MICU2 lowers the required Ca^2+^ concentration in the intermembrane space for MICU1-regulated MCU activation, resulting in increased mitochondrial Ca^2+^ uptake [52]. On the other hand, ablation of MICU1 prevents indirect binding of MICU2 to the MCU, resulting in unregulated Ca^2+^ entry into the mitochondria [61,62]. Studies with MICU1 knockout as well as with MICU1 knockdown have described an inverse relationship between extra-mitochondrial Ca^2+^ concentrations and mitochondrial Ca^2+^ uptake [62,63]. This suggests that the absence of MICU1 at low/resting extra-mitochondrial Ca^2+^ concentrations leads to increased mitochondrial Ca^2+^ uptake, while high extra-mitochondrial Ca^2+^ concentrations are associated with decreased mitochondrial Ca^2+^ uptake. The final subunit, which has been proposed to contribute to the structure of the MCU complex is a scaffolding factor called mitochondrial calcium uniporter regulator 1 (MCUR1), which can interact with the MCU but not with MICU1 [64]. However, there are two possible interpretations of the direct or indirect role of MCUR1 in modulating mitochondrial Ca^2+^ uptake. Several studies have demonstrated a regulatory effect of MCUR1 on MCU complex activity, noting reduced mitochondrial Ca^2+^ uptake in the absence of MCUR1 and a decrease in reducing equivalents as well as ATP generation [65,66,67]. Alternatively, MCUR1 has been suggested to have a potential role as a Complex IV assembly factor in the mitochondrial respiratory chain [68]. Interestingly, the relative abundance of distinct MCU components has been reported to be different in distinct cell types or tissues [35], and it has been proposed that the specific stoichiometry of the MCU subunits may define the functional characteristics of the channel, including Ca^2+^ permeation across the pore, the activation threshold and the cooperativity. Consistent with this hypothesis, it has been reported that in parallel with the distinct relative abundance of MCU components [35], MCU activity also varies greatly among cell types [69]. Although the stoichiometry of the different MCU subunits and their relative abundance in different tissues, including in the pancreatic β-cell, are largely unknown, we tentatively speculate that these cells may have a specific configuration of the MCU that reduces the Ca^2+^ activation threshold.

**Figure 2:**
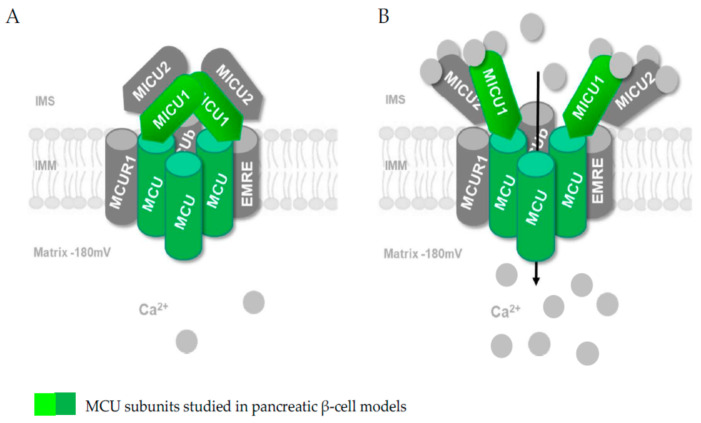
The mitochondrial calcium uniporter (MCU) comprises distinct pore-forming (MCU/MCUb) and associated proteins (essential MCU regulator (EMRE), mitochondrial calcium uptake (MICU) 1, MICU2, MICU3 and mitochondrial calcium uniporter regulator 1 (MCUR1)). The subunits that have already been studied in models of the pancreatic β-cell are rendered in green. (**A**) Model of the closed conformation of MCU. (**B**) Following glucose stimulation, the entry of Ca^2+^ into the matrix space is mediated by MCU. IMM, inner mitochondrial membrane; IMS, intermembrane space.

Pancreatic β-cells have been demonstrated to express a functional MCU, and some studies have investigated the role of MCU subunits on insulin secretion [15,16,17] (Figure 2).

Not surprisingly, the existence of MCU and MICU1 subunits in pancreatic β-cell models has been reported and functionally validated (see Section 4) [15,16,17,55]. In addition, a study recently published as a preprint in bioRxiv but not yet peer-reviewed proposes the existence of MICU2 subunits in rat and human insulin-secreting cell lines as well as in mouse islets (Mitochondrial Clearance of Ca^2+^ Controls Insulin Secretion; https://doi.org/10.1101/830323 (accessed on 1 February 2021); Vishnu, Hamilton, Bagge, Wernersson, Cowan, Barnard, Sancak, Kamer, Spégel, Fex, Tengholm, Mootha, Nicholls and Mulder). Further studies are expected to consolidate the specific role of the other subunits of the MCU in the pancreatic β-cell (Figure 2) and fully define the structure of this transporter. However, the crucial role of the MCU complex in the mitochondrial Ca^2+^ transport of the pancreatic β-cell and in the pancreatic β-cell metabolism–secretion coupling is already well established and will be discussed in the next section.

## 4. Role of Mitochondrial Ca^2+^ Uptake in the Pancreatic β-Cell

The importance of mitochondrial Ca^2+^ in pancreatic β-cell function was efficiently highlighted by modulating mitochondrial Ca^2+^ via increased matrix Ca^2+^ buffering capacity, achieved by targeting exogenous Ca^2+^-binding proteins into the matrix [13] or by loading the cells with the Ca^2+^ chelator BAPTA (1, 2-bis(2-aminophenoxy) ethane-N, N, N′, N′-tetraacetic acid) [70]. The impaired bioenergetic response to elevated glucose recorded in these two studies revealed the physiological importance of mitochondrial Ca^2+^ in energy metabolism required for signal-dependent hormone secretion. The discovery of the molecular nature of the mitochondrial Ca^2+^ uniporter [14,40] then enabled clarification of the role of mitochondrial Ca^2+^ in pancreatic β-cell function and opened new perspectives for pharmacological and nutritional interventions. In murine pancreatic β-cells, knockdown of MCU showed a strong reduction in mitochondrial Ca^2+^ uptake, accompanied by impaired ATP production [17], consistent with a role of mitochondrial Ca^2+^ in metabolism–secretion coupling. Similar results were obtained in pancreatic β-cell lines (INS-1 and INS-1E cells). Genetic depletion of two subunits of the MCU complex (MCU and MICU1) was characterized by reduced hyperpolarization of the inner mitochondrial membrane, diminished mitochondrial Ca^2+^ transients, impaired respiration rate and ATP levels, and reduced insulin secretion during glucose stimulation [15,16]. Taken together, these results demonstrate the importance of mitochondrial Ca^2+^ for signal transduction in β-cells and the impact of the MCU on signal-dependent hormone secretion.

The crucial role of mitochondrial Ca^2+^ in glucose-induced insulin secretion was finally validated by Georgiadou and collaborators in a mouse model, in which MCU was highly selectively deleted in pancreatic β-cells [55]. In this paper, the authors showed that mitochondrial Ca^2+^ uptake, glucose-induced ATP production and insulin secretion were substantially impaired in their animal model in vitro. In the living β-cell-specific MCU-null mice, the first phase of insulin release was also impaired, despite paradoxical improvements in systemic glucose tolerance. Despite the apparent compensatory mechanisms proposed to explain the maintained glucose tolerance in β-cell-specific MCU-null mice (which remain to be established), this study indicated that agents affecting mitochondrial Ca^2+^ uptake in the pancreatic β-cell may alter insulin secretion and diabetes risk.

The molecular characterization of the MCU is shedding light on the role of this transporter in pancreatic β-cell function; however, the precise mechanism and contribution of mitochondrial Ca^2+^ to the regulation of insulin secretion is not fully understood. Consistent with the universal role of mitochondrial Ca^2+^ in the regulation of cellular energetics [12,71], an essential role of mitochondrial Ca^2+^ for effective ATP generation in pancreatic β-cells is well established [18]. Mitochondrial Ca^2+^ is required for the activation of matrix dehydrogenases involved in pyruvate metabolism and the TCA cycle [18,72]. Mitochondrial Ca^2+^-activated dehydrogenases form reducing equivalents in the mitochondrial matrix, promoting the reduction of mitochondrial redox pairs. Most notably, studies in Bristol in the 1960s and 1970s led to the recognition that mitochondrial Ca^2+^ promotes the supply of reducing equivalents in the form of NADH or FADH2 [73,74,75]. Four Ca^2+^-activated mitochondrial dehydrogenases were specifically described: FAD-glycerol phosphate dehydrogenase (located on the outer surface of the inner mitochondrial membrane and influenced by changes in cytoplasmic Ca^2+^ concentration), pyruvate dehydrogenase, NAD-isocitrate dehydrogenase and oxoglutarate dehydrogenase (the latter three are localized within the mitochondria and are regulated by changes in mitochondrial matrix Ca^2+^ concentration). Following early studies with isolated mitochondria, the effects on Ca^2+^ regulation of mitochondrial metabolism were confirmed in situ [71]. In the pancreatic β-cell, the mitochondrial Ca^2+^ level reached during glucose-induced cell activation (see Section 3) was sufficient to stimulate matrix dehydrogenases. Therefore, the K_0.5_ of pyruvate dehydrogenase for Ca^2+^ activation is about 1 µM and the K_0.5_ of oxoglutarate dehydrogenase is in the range of 0.2 to 2 µM, depending on the ATP/ADP levels [76]. In this context, we proposed to extend this bioenergetics and dehydrogenase-dependent role of mitochondrial Ca^2+^ by discovering the significant contribution of the ATP-synthase-dependent mitochondrial respiration [77]. According to our model, in the pancreatic β-cell, the cytosolic Ca^2+^ rise during glucose stimulation affects mitochondrial activity. Mitochondria take up and release Ca^2+^ ions, leading to a transient increase in matrix Ca^2+^. These mitochondrial Ca^2+^ signals accelerate oxidative metabolism and simultaneously stimulate ATP-synthase-dependent respiration. Coordinated activation of these two processes allows the respiratory rate to change several-fold with only small alterations in the NAD(P)H:NAD(P)^+^ ratio (the ratio between the reduced and oxidized form of the nicotinamide adenine dinucleotide, or of the nicotinamide adenine dinucleotide phosphate), promoting robust insulin secretion [77]. Consistent with the proposition that mitochondrial Ca^2+^ uptake is a critical event in cellular bioenergetics and thus in the metabolic coupling of insulin, specific buffering or suppression of the mitochondrial Ca^2+^ increase lowers glucose-induced respiration and ATP synthesis, and impairs second-phase insulin secretion [13,17].

An additional mechanism linking mitochondrial Ca^2+^ to signal transduction in β-cells is related to the ability of mitochondria to take up and buffer intracellular Ca^2+^ and to affect the tone and frequency of cytosolic Ca^2+^ oscillations [18]. This regulation may contribute to control the pulsatility of insulin release [78]. The ability of mitochondria to buffer cytosolic Ca^2+^ has also recently been linked to the regulation of the insulin secretion through local Ca^2+^ buffering at the sub-plasma membrane level [79]. In this study, linking mitochondrial localization with mitochondrial Ca^2+^ and β-cell function, the authors proposed that changes in mitochondrial distribution may be important for the generation of the Ca^2+^ microdomains required for efficient insulin granule release. The precise contribution of mitochondrial Ca^2+^ rise to the shaping of cytosolic Ca^2+^ signaling is not completely established in β-cells. However, the aforementioned preprint in bioRxiv, which has not yet been peer-reviewed, (Mitochondrial Clearance of Ca^2+^ Controls Insulin Secretion; https://doi.org/10.1101/830323; Vishnu, Hamilton, Bagge, Wernersson, Cowan, Barnard, Sancak, Kamer, Spégel, Fex, Tengholm, Mootha, Nicholls and Mulder), seems to shed some light on this function. Thus, by ablating MICU2 subunits of the MCU complex in insulin-secreting rat and human cell lines as well as in mouse islets, the authors reported reduced glucose-induced mitochondrial Ca^2+^ elevation and impaired bioenergetics and insulin secretion. In MICU2-deficient cells, although KCl-evoked sub-plasmalemmal Ca^2+^ increases were more pronounced, the global cytosolic Ca^2+^ response was surprisingly reduced. The authors concluded that MCU plays a role in stimulated β-cells by regulating net Ca^2+^ entry across the plasma membrane. They proposed that this was linked to the clearing of sub-plasmalemmal Ca^2+^ levels by mitochondria located near the plasma membrane.

In addition to the regulation of bioenergetics and the modulation of cytosolic Ca^2+^, mitochondrial Ca^2+^ has also been proposed to modulate β-cell function, linked to the activation of the mitochondrial permeability transition pore (PTP) [80]. The PTP is a large mitochondrial inner membrane channel responsible for the so-called mitochondrial permeability transition, which is a mitochondrial Ca^2+^-dependent, redox-dependent and cyclosporine-A-inhibited permeabilization of the inner mitochondrial membrane [81,82]. The opening of the PTP plays an important role in the physiopathology of several tissues, including pancreatic β-cells [83,84,85,86,87]. Given the crucial role of the PTP in cellular physiopathology and its mitochondrial Ca^2+^-dependence [88], the modulation of this channel provides an additional link between mitochondrial Ca^2+^ signaling and the function and fate of pancreatic β-cells.

The molecular identity of the PTP is still a matter of debate [81,89,90,91]; however, the protein cyclophilin D has been demonstrated to be a regulator of the PTP [92,93]. Therefore, the PTP inhibitor cyclosporine-A prevented PTP activation in wild-type mice but not in cyclophilin D knockout animals, demonstrating that cyclophilin D is a regulatory subunit of the PTP and represents the target for PTP inhibition by cyclosporine A. Although the specificity of cyclosporine A has been criticized, several pharmacological cyclosporine A-based studies in pancreatic β-cells proposed the existence of the mitochondrial permeability transition in this cell type [85,87,94]. These manuscripts emphasized the importance of the PTP for the secretory function of β-cells [83,85], and as a common effector of both apoptosis and necrosis [94,95].

In two semi-permeabilized pancreatic β-cell lines (MIN6 and INS-1), the existence of both a mitochondrial Ca^2+^-induced and thiol cross-linking-dependent, and a mitochondrial Ca^2+^-independent mitochondrial permeability transition has been reported [85]. Inhibition of PTP opening with cyclosporine A suppressed glucose-induced insulin secretion [83,85]. In another study, cyclosporine A decreased Pdx1 deficiency-induced cell death in mouse insulinoma MIN6 cells by preventing PTP opening [84]. These results were confirmed in a genetic mouse model after ablation of Ppif, the gene encoding cyclophilin D. Disruption of this gene restored β-cell mass, reduced β-cell death, and normalized fasting blood glucose and glucose and insulin responses to an acute glucose challenge in adult mice previously kept on a high-fat diet [84]. The authors concluded that the PTP is a critical regulator of pancreatic β-cell death. Another study showed that cyclosporine A prevented PTP opening and protected against both high-dose glucose- and fructose-induced cell death [96]. In addition, it has been suggested that pharmacological inhibition of the PTP during islet transplantation may improve islet cell survival and graft success [86].

In summary, the mitochondrial Ca^2+^-induced PTP may play two distinct roles in the physiology and pathology of the pancreatic β-cell. While activation of the PTP is required to promote insulin secretion upon glucose stimulation [83,85], PTP inhibition protects against glucotoxicity as well as hypoxia and substrate deficiency during islet transplantation [86,96]. Given the dependence of the PTP on mitochondrial Ca^2+^ levels, MCU activators are expected to promote insulin secretion; conversely, MCU inhibitors are potential agents to protect β-cells during islet transplantation or nutrient stress in vivo [97]. Importantly, a large number of inhibitors of mitochondrial Ca^2+^ transport have been synthesized recently [97,98,99,100]. However, MCU inhibitors can non-specifically affect not only different cellular transport systems of Ca^2+^ but also various systems in the mitochondria. For instance, the new penetrating inhibitor of the Ca^2+^ uniporter, DS16570511, has multiple effects on the mitochondria of cerebral cortex cells, while stimulating cellular survival during glutamate overload [101]. Thus, a comprehensive analysis of the mechanisms of action of mitochondrial Ca^2+^ transport modulators is crucial to conclusively establish the potential effects of these compounds on pancreatic β-cell function.

## 5. Mitochondrial Ca^2+^ Extrusion in the Pancreatic β-Cell

During cell stimulation, the amplitude and duration of mitochondrial Ca^2+^ elevation reflect the balance between uptake and release mechanisms [11,102,103]. Many studies have focused on MCU-mediated mitochondrial Ca^2+^ uptake in different systems, including the pancreatic β-cell. However, mitochondrial Ca^2+^ extrusion has also been demonstrated to play a key role in cellular physiopathology and signaling [64,102,104]. Therefore, prolonged (pathological) accumulation of Ca^2+^ in the matrix space can lead to mitochondrial Ca^2+^ overload, followed by opening of the mitochondrial permeability transition pore [88,89], resulting in the activation of cell death signals. To avoid this transition from the stimulatory to detrimental effects of Ca^2+^, mitochondria possess two membrane systems to extrude Ca^2+^: the mitochondrial Na^+^/Ca^2+^ exchanger and the mitochondrial H^+^/Ca^2+^ exchanger [64,102,104,105].

The molecular identity of the mitochondrial Na^+^/Ca^2+^ exchanger was revealed in 2010 and attributed to the protein NCLX [104]; subsequently, the impact of NCLX on pancreatic β-cell function was investigated [106]. The existence and role of a mitochondrial Ca^2+^/H^+^ exchanger in the pancreatic β-cell is a more complicated topic. Therefore, the protein LETM1 was proposed as a high-affinity mitochondrial Ca^2+^/H^+^ exchanger, capable of driving both the extrusion and uptake of Ca^2+^ into energized mitochondria at sub-micromolar Ca^2+^ concentrations [107,108]. Interestingly, LETM1 is expressed in the β-cell line INS-1 and it has been proposed to contribute to mitochondrial Ca^2+^ sequestration (but not extrusion), depending on the source and mode of mobilized Ca^2+^ [15]. Therefore, mitochondrial Ca^2+^ sequestration of Ca^2+^ that entered the INS-1 cells via depolarization-activated L-type Ca^2+^ channels of the plasma membrane was blunted by diminution of LETM1 expression [15]. However, silencing LETM1 in INS-1 cells did not attenuate mitochondrial sequestration of intracellularly released Ca^2+^. The authors concluded that these data point to LETM1 as a high-affinity Ca^2+^ carrier [15]. However, LETM1 was previously indicated to mediate K^+^/H^+^ exchange in the mitochondrial inner membrane [109,110]. In addition, exogenous LETM1 expression led to a direct increase in K^+^-induced proton extrusion, whereas mitochondrial Ca^2+^ efflux was not altered [64]. Other studies support a role of LETM1 as a K^+^/H^+^ exchanger [111,112]. Moreover, they indicate a key role of LETM1 in monovalent cation homeostasis and suggest an indirect effect of LETM1 on the modulation of mitochondrial transmembrane Ca^2+^ fluxes, which can reflect the effects on Na^+^/H^+^ exchange activity [111]. In summary, the existence and role of a mitochondrial Ca^2+^/H^+^ exchanger in the pancreatic β-cell remains to be elucidated, and additional studies are needed to reach a conclusion.

**Figure 3:**
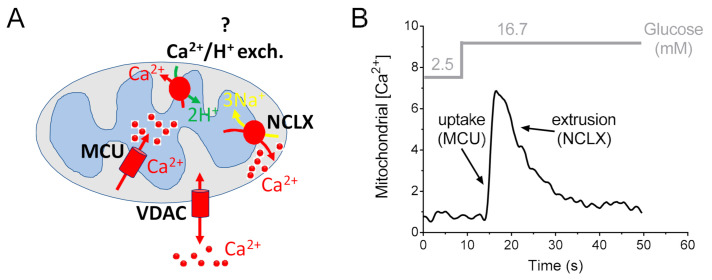
Contribution of mitochondrial Ca^2+^ uptake and mitochondrial Ca^2+^ extrusion to mitochondrial Ca^2+^ signaling in the pancreatic β-cell. (**A**) Ca^2+^ transport proteins of pancreatic β-cell mitochondria. In the outer mitochondrial membrane, the voltage-dependent anion channel VDAC facilitates the diffusion of Ca^2+^, ions and other small solutes. However, mitochondrial Ca^2+^ uptake is mainly regulated at the level of the inner membrane. Therefore, in β-cell mitochondria, the influx of Ca^2+^ into the matrix is mediated by the Ca^2+^-selective channel MCU complex; Ca^2+^ is then extruded by the Na^+^/Ca^2+^ exchanger NCLX. The existence and the molecular identity of a pancreatic β-cell mitochondrial Ca^2+^/H^+^ exchanger has not yet been proven and is here highlighted with a question mark (?) (see text for details). (**B**) Example of a calibrated trace of mitochondrial Ca^2+^ elevation in the pancreatic β-cell line INS-1E stimulated with glucose, as indicated (gray). The uptake and release phases of the mitochondrial Ca^2+^ are highlighted. The transporters which have been demonstrated to substantially contribute to the uptake and the release are MCU and NCLX, respectively. To calibrate mitochondrial Ca^2+^, INS-1E cells were transfected with the genetically encoded mitochondrial luminescence Ca^2+^ sensor aequorin, which is reported to overestimate the average Ca^2+^ rise in cellular compartments [113].

Conversely, convincing data on the molecular identity and function of the mitochondrial Na^+^/Ca^2+^ exchanger in the pancreatic β-cell have been reported (Figure 3).

Prior to the discovery of the molecular identity of the mitochondrial Na^+^/Ca^2+^ exchanger NCLX, previous studies used the mitochondrial Ca^2+^ exchanger inhibitor CGP-37157 to inhibit this transporter [114]. This treatment resulted in enhanced mitochondrial oxidative metabolism, ATP production and insulin secretion in rat pancreatic islets [114]. In contrast, other observations suggested that the use of the inhibitor CGP-37157 did not lead to enhanced glucose-dependent ATP production [115]. The major complication is that the CGP-37157 inhibitor is not specific and may interact with other Ca^2+^ transport pathways in the β-cell, including cytosolic Ca^2+^ signaling (by blocking the L-type Ca^2+^ channels, LTCC) [116], sarcoplasmic/endoplasmic reticulum Ca^2+^-ATPase (SERCA pumps) and ryanodine receptors (RyR) [117].

The molecular discovery of NCLX allowed us to highlight the importance of mitochondrial extrusion in different tissues [64,104,105], including the pancreatic β-cell [106,118]. NCLX is expressed in the mitochondria of pancreatic β-cells, where it mediates mitochondrial Ca^2+^ extrusion [106,118]. By silencing NCLX using small interfering RNA, Nita and collaborators demonstrated that NCLX promotes mitochondrial Ca^2+^ extrusion from β-cells in MIN6 and in primary β-cells after glucose stimulation, and modulates both basal mitochondrial membrane potential and resting calcium levels. In addition, NCLX activity plays a major role in controlling both the rate and amplitude of cytosolic Ca^2+^ responses [106]. Surprisingly, NCLX had a small effect on high glucose-dependent ATP production but primarily regulated the rate of glucose-dependent insulin secretion, particularly during the first phase of insulin secretion [106]. Thus, the authors argued against a major energetic role for the exchanger in Ca^2+^ signaling linked to insulin secretion. They proposed that the Ca^2+^ transport activity mediated by NCLX and its strong effect on increasing cytosolic Ca^2+^ responses are the primary roles of NCLX in the context of insulin secretion. They suggested a model of mitochondrial Ca^2+^ efflux that modulates pancreatic β-cell function via NCLX-dependent shaping of the cytosolic glucose-dependent Ca^2^^+^ response, which may regulate the rate of insulin secretion. However, further in vivo studies with transgenic NCLX knockout mice are required to determine the precise contribution of mitochondrial Ca^2+^ extrusion to β-cell bioenergetics, Ca^2^^+^ homeostasis and islet physiology.

## 6. Mitochondrial Ca^2+^-Targeted Intervention Strategies to Modulate Pancreatic β-Cell Function

Given the importance of mitochondrial Ca^2+^ for pancreatic β-cell signal transduction and the possibility of modulating mitochondrial Ca^2+^ with natural bioactives [119], we recently investigated the effects of a mitochondrial Ca^2+^-targeted nutritional intervention strategy on metabolism–secretion coupling in a model of pancreatic insulin-secreting cells (INS-1E) [45]. We discovered that acute treatment of pancreatic INS-1E cells with the natural plant flavonoid and MCU activator kaempferol [119] increased glucose-stimulated mitochondrial Ca^2+^ elevation, which potentiated insulin secretion. Conversely, the MCU inhibitor mitoxantrone inhibited mitochondrial Ca^2+^ uptake and prevented both glucose-induced insulin secretion and kaempferol-potentiated effects. Kaempferol-dependent potentiation of insulin secretion was also validated in a model of standardized human pancreatic islets. We concluded that a mitochondrial Ca^2+^-targeted nutritional intervention activated metabolism–secretion coupling in insulin-secreting cells by modulating mitochondrial Ca^2+^ uptake.

Although pharmacological inhibition of the kaempferol-induced effect obtained by the mitochondrial Ca^2+^ inhibitor mitoxantrone [97] indicates a certain level of causality between mitochondrial Ca^2+^ modulation and β-cell function, it has not yet been definitively proven whether the modulation of mitochondrial Ca^2+^ by kaempferol is a direct or indirect effect of this polyphenol on the MCU complex.

In another intervention study, we demonstrated that the natural bioactive quinic acid increases glucose-stimulated mitochondrial Ca^2+^ rise, which enhanced insulin secretion in both INS-1E cells and mouse islets, and improved glucose tolerance in mice [120]. In this study, the naturally occurring polyol quinic acid was not a direct activator of MCU, but it enhanced the release of Ca^2+^ from the endoplasmic reticulum, thereby improving Ca^2+^ transfer between the endoplasmic reticulum and mitochondria. This transient mitochondrial Ca^2+^ increase was accompanied by the activation of two mitochondrial Ca^2+^-dependent processes (oxidative metabolism and ATP synthase-dependent respiration), which coordinately promoted sustained insulin secretion in the pancreatic β-cell [77].

Although preclinical and clinical evidence, including interventions in genetic MCU-ablated models, is still needed to validate the efficacy and safety of these phytochemical-based interventions, these studies suggest that bioactive agents that increase mitochondrial Ca^2+^ in pancreatic β-cells could be used to treat diabetes.

## 7. Conclusions

β-cell mitochondria contain a variety of ion channels in both the inner and the outer mitochondrial membrane, with important roles in stimulus–secretion coupling and cell viability [21]. The role of mitochondrial ion channels, including the MCU, in the β-cell has only partly been elucidated and is nowadays underestimated. The molecular definition of the MCU and its role in pancreatic β-cell signal transduction opens the possibility of developing a mitochondrial Ca^2+^-targeted intervention strategy for β-cell health and, prospectively, for diabetes treatment. Stimulation of the MCU has been demonstrated to improve β-cell energy metabolism during nutrient stimulation. Such a mechanism could explain how MCU activators might have a beneficial effect on stimulus–secretion coupling in β-cells, leading to improved glucose homeostasis. Encouraging results with the natural bioactive MCU activator kaempferol indicate the potential beneficial effects of the MCU-targeted strategy on pancreatic β-cell function. However, kaempferol is a non-specific activator of mitochondrial Ca^2+^ rise in pancreatic β-cells. A more specific MCU-targeted pharmacology is expected to improve MCU activation of the pancreatic β-cell, promoting beneficial effects in the context of diabetes treatment.

New studies targeting the proteins that control mitochondrial Ca^2+^ uptake should reveal whether altered insulin secretion is causally related to diabetes progression and could potentially expand the repertoire of therapeutic tools to treat this disease.

## Data Availability

No new data were created or analyzed in this study. Data sharing is not applicable to this article.
